# Open Access Publishing – A New Path

**Published:** 2009-06-16

**Authors:** Neil Smalheiser, Sandra L De Groote, Mary M Case

**Affiliations:** 1 UIC Psychiatric Institute MC912, University of Illinois at Chicago, 1601 W. Taylor Street, room 525 Chicago, IL 60612; 2 University Library, University of Illinois at Chicago, 801 S Morgan, Chicago, IL 60607

## Editorial

The open-access movement in journal publishing has generally been equated with the author-pays model, and contrasted with the subscription or reader-pays model. Yet there is a third possible path that has received scant public attention: For example, at the University of Illinois at Chicago, the Library system is hosting several peer-reviewed, open-access journals that do not charge any fees from authors or readers. These include First Monday, a leading information-science journal, and Journal of Biomedical Discovery and Collaboration, a multi-disciplinary journal that is indexed in PubMed. Virginia Polytechnic Institute also hosts a variety of free peer-reviewed open-access journals (http://scholar.lib.vt.edu/), and many journals produced using Open Journal Systems software fall into this category as well (http://pkp.sfu.ca/ojs-journals/). 

The actual costs of supporting online journals are surprisingly modest - for minimal costs one can host servers providing open access journal management software and layout software, resources that can be shared by all journals in the same system. The efforts of editors and peer reviewers are non-compensated, as is true even in commercial ventures. Copyeditors can be hired to edit articles for spelling, grammar and journal style, but this is optional -- note that PLOS ONE and most Biomed Central journals charge article fees yet do not provide copyediting services for authors. 

It is natural for institutional libraries to take a leading position in open-access publishing, since they bear the brunt of skyrocketing subscription fees under the reader-pays model. Prospective authors should be encouraged to consider publishing in the free open-access journals that already exist – and editors launching new journals should consider the library-hosted model as an ideal way forward. 

